# Editorial: Harnessing Useful Rhizosphere Microorganisms for Pathogen and Pest Biocontrol - Second Edition

**DOI:** 10.3389/fmicb.2019.01935

**Published:** 2019-08-28

**Authors:** Aurelio Ciancio, Corné M. J. Pieterse, Jesús Mercado-Blanco

**Affiliations:** ^1^Istituto per la Protezione Sostenibile delle Piante, Consiglio Nazionale delle Ricerche, Bari, Italy; ^2^Department of Biology, Science4Life, Utrecht University, Utrecht, Netherlands; ^3^Instituto de Agricultura Sostenible, Agencia Estatal Consejo Superior de Investigaciones Científicas, Córdoba, Spain

**Keywords:** biocontrol, induced resistance, plant growth promotion, rhizosphere microbiology, plant microbe interaction soil microbiology

There is a worldwide interest in the exploitation of beneficial plant-associated microorganisms as an alternative to pesticides for pest and disease management. It is underpinned by practical and social reasons, including safety of consumers, farmers, and field workers, as well as the need for sustainable practices safeguarding the environment and protecting its biodiversity. Cost of conventional pesticides and the insurgence of resistance in pests also re-direct farmers' choice toward safer approaches. This trend is observed also in fast-growing population economies, propelling the global demand for eco-sustainable technologies.

Understanding the role of rhizosphere microorganisms in the control of pests and diseases appears as a growing research field, as shown by the sharp increase of studies carried out during the period 2000–2019. The number of records retrieved through a Google Scholar query with keywords “microorganisms,” “control,” “pest,” and “diseases” increased from around 5000 (2000–2005) to ~8500 and >20,000 (2006–2010 and 2011–2019, respectively), when the term “rhizosphere” was added. Without the latter the records instead lowered from around 17,000 to ~15,000 in the last period (interrogation dated August 2, 2019). However, in spite of this increased interest in rhizosphere ecology managing and exploiting living organisms to regulate or control other noxious species still remains a complex task. Detailed data on interacting variables and processes are needed, as their final result often differs significantly from the simple sum of effects. Any information boosting our capacity to solve problems related to safer plant protection is, therefore, more than welcome.

## Biocontrol of *Phytophthora*

The Oomycete *Phytophthora infestans*, the most widespread and severe pathogen of potato, is a global threat due to its capacity to develop new genotypes overcoming the resistance present in many cultivars (Fry, 2008; Cooke et al., 2011). Rhizo/phyllosphere *Pseudomonas* strains have been tested against this pathogen. The potato cultivar to which bacteria were originally associated, and the combined use of strains were found to underpin beneficial effects, including a higher disease inhibition (De Vrieze et al.). These authors tested the rhizosphere complexity, with combinations of native bacteria yielding benefits, not previously inferred from *in vitro* data. This approach showed that multiple strains can be exploited in combination, using poorly competitive bacteria that do not interfere each other. Results stressed the potential of communities based on native microorganisms rather than single isolates, inhibiting *P. infestans* or halting its progression.

In a similar approach, Caulier et al. screened *Bacillus* and *Pseudomonas* spp. strains against potato pathogens including *P. infestans*. The most significant antagonistic activity appeared to be related to production of metabolites such as bacilysin, bio-surfactants, and siderophores, characterizing some *Bacillus* spp. strains. Data indicated that indigenous rhizosphere microflora may yield effective bio-management tools.

## Biocontrol of Fungal Diseases

The exploitation of rhizosphere microorganisms for management of fungal diseases is a fertile research field (Toyota and Shirai, 2018). Species mostly include antagonistic fungi such as *Trichoderma* spp. and bacteria of genera *Pseudomonas, Bacillus*, and *Streptomyces*. As assays showed biocontrol efficacy for many isolates, there is a need for characterizing metabolic and molecular mechanisms underpinning these activities.

### Trichoderma

The role of gene regulatory elements may reveal key clues in biocontrol agents. In *Trichoderma harzianum* the transcription factor THCTF1 is involved in the production of 6-pentyl-pyrone, a volatile secondary metabolite active in interspecies cross-talk. THCTF1A is a homolog of the multiprotein bridging factor 1 (*mbf1*). Expression and regulation of both genes appeared to be important in modulating the activity of the fungus. Transformants overexpressing *Thmbf1* were used to investigate the effect of this gene in biocontrol of *Fusarium oxysporum* f. sp. *lycopersici* race 2 (FO) and *Botrytis cinerea*. *Thmbf1* affected production of volatile organic compounds (VOC) and low molecular weight molecules by *T. harzianum* with antifungal activity against FO. *Thmbf1* overexpression negatively affected *T. harzianum* biocontrol efficacy against both pathogens on susceptible tomato (Rubio et al.).

Manganiello et al. investigated transcriptomic and metabolomic changes induced by a *T. harzianum* strain, or its secondary metabolite harzianic acid, in Micro-Tom tomato infected by the soil-borne pathogen *Rhizoctonia solani*. Genes involved in resistance to biotic stress were differentially expressed, confirming *T. harzianum* ability to activate a defense response in plants. Genes of the ethylene/jasmonate and salicylic acid signaling pathways were up-regulated, as well as genes involved in reactive oxygen species (ROS) detoxification. Harzianic acid induced expression of defense genes such as protease inhibitors, CC-NBS-LRR resistance proteins, and hormone interplay. Both treatments increased steroidal glycoalkaloids, confirming activation of metabolic chemical defenses.

### Pseudomonas

Rhizosphere competence is a key factor for a successful biocontrol agent, as plant species, soil type, and pathogen types affect the microbial community composition in the rhizosphere (Schreiter et al., 2014). The biocontrol efficacy of a *Pseudomonas* sp. isolate against *R. solani* on potato and lettuce was tested in three soil types. The antagonist colonized the rhizosphere of both crops and reduced the severity of black scurf on potato and bottom rot on lettuce in all soils. The *Pseudomonas* sp. isolate differently affected the bacterial community composition, depending on the host plant, as its effect was more evident in lettuce than potato (Schreiter et al.).

*Pseudomonas* spp. and antimicrobial metabolites such as 2,4-diacetylphloroglucinol (DAPG) or phenazines (PHZ), are likely involved in the insurgence of disease-suppressive soils, inhibiting soilborne plant diseases. However, antimicrobial-producing species can be found also in disease-conducive soils. Imperiali et al. compared soils from Swiss cereal farms, considering their resistance against two soilborne pathogens, the abundance of *Pseudomonas* spp. the biosynthesis DAPG, PHZ, and pyrrolnitrin, and the ability to support their production on roots. All these traits did not correlate to soil disease resistance. Data showed that this capacity depended on the pathogen species, as shown by soils exclusively not conducive for *Gaeumannomyces tritici* or *Pythium ultimum*. Factors related to soil structure and availability of nutrients were found to affect abundance and production of antimicrobial compounds.

A *Pseudomonas* spp. strain was tested for management of blast disease, caused by *Pyricularia grisea*, on the finger millet *Eleusine coracona* “Ragi,” a cereal used in semi-arid regions of Asia and Africa. It inhibited *P*. *grisea*, solubilized phosphate and produced antifungal metabolites, siderophores, hydrolytic enzymes, and indolacetic acid. The strain had a direct effect on the pathogen, causing hyphal deformation, with a minimum impact on root microbial biomass and other rhizobacteria. It also reduced blast infestation and enhanced plant vigor and growth, showing a prolonged shelf-life when stored as a liquid formulation (Sekar et al.).

Similarly, several isolates collected by Gómez-Lama Cabanás et al. from olive rhizosphere showed potential against Verticillium wilt of olive, caused by *Verticillium dahliae*. Three *Pseudomonas* spp. strains were selected (PIC25, PIC105, and PICF141) with high *in vitro* inhibition against the pathogen. Effectiveness against the defoliating pathotype of *V. dahliae* was demonstrated, in particular for PICF141. Molecular analyses showed PICF141 as closest to *P. lini* (“*Pseudomonas mandelii* subgroup,” within the “*Pseudomonas fluorescens* group”). Strains PIC25 and PIC105 were affiliated to the “*Pseudomonas aeruginosa* group,” with *P. indica* as the closest relative. The isolates showed genotypic and phenotypic traits associated with plant growth promotion and/or biocontrol, including production of phytase, xylanase, catalase, cellulase, chitinase, glucanase activities, siderophores, and HCN. *Pseudomonas indica* PIC105 was identified for the first time as a biocontrol agent of fungi. Root colonization was useful for their application in biocontrol formulations.

### Bacillales

Several *Bacillus* species induce beneficial effects on plants through the production of antimicrobial compounds, as shown by species with growth-promotion and/or biocontrol activities such as the model strain *Bacillus velezensis* FZB42. Genes involved in synthesis of secondary metabolites, suppressing the growth of pathogens and inducing systemic resistance, were found together with VOC involved in biocontrol (Fan et al.).

*Bacillus amyloliquefaciens* strain JCK-12, isolated from soil, showed a significant antifungal activity vs. *F. graminearum*, the causal agent of Fusarium head blight. The fungus dramatically affects yields, with high risks to human and animal health due to its mycotoxins. JCK-12 fermentation broth and formulations reduced the disease incidence in wheat, likely related to production of cyclic lipopeptides including iturin A, fengycin, and surfactin. Iturin A inhibited *F. graminearum* spore germination, whereas the other lipopeptides showed, when mixed with iturin A, only a synergistic inhibitory effect on germination. Strain JCK-12 also affected production of trichothecenes. Moreover, it showed synergistic antifungal effects with synthetic fungicides *in vitro*, controlling the disease in greenhouse and field conditions (Kim et al.).

Lipopeptides production is a beneficial trait displayed by some biocontrol bacteria (Velho et al., 2011). Surfactin, bacillomycin, and fengycin, detected in cultures of *B. methylotrophicus* (syn. of *B. velezensis*) by mass spectrometry, inhibited *in vitro* growth of the necrotrophic plant pathogen *B. cinerea*. Electron microscopy observations confirmed this effect showing an alteration of the fungus morphology upon the interaction with lipopeptides, with degeneration of cell organelles and production of early stage resistance structures. Assays with tomatoes, grapes, and strawberries confirmed the lipopeptides efficacy against *B. cinerea*, with the induction of an antioxidant activity in fruit (Toral et al.).

*Paenibacillus polymyxa* (former *B. polymyxa*) strain HY96-2 is a plant-growth-promoting rhizobacterium and a biocontrol agent, commercialized in China as a microbial biopesticide. Comparison of complete genome sequence data with other *P. polymyxa* strains indicated the potential to antagonize plant diseases by biofilm formation, antibiotic production, and induced systemic resistance (Luo et al.).

### Streptomyces

Several *Streptomyces* spp. are effective biocontrol agents in different climatic conditions and agroecosystems. A *S. globosus* isolate, originating from healthy date palm rhizosphere in the United Arab Emirates, showed antagonism vs. *Thielaviopsis punctulata*, the causal agent of black scorch disease. Its activity was associated *in vitro* with production of diffusible antifungal metabolites that inhibited *T. punctulata* mycelial growth. Greenhouse and pathogenicity tests showed that the isolate suppressed black scorch disease and prevented its spread at a level comparable to that of a synthetic fungicide (Saeed et al.). In the same country, a selective *in vitro* screening showed two *Streptomyces* spp. and one *Micromonospora* sp. isolates with a strong inhibitory effect against mango dieback, caused by the fungus *Lasiodiplodia theobromae*. In particular, activity of *S. samsunensis* was related to antibiosis and production of cell-wall-degrading enzymes (CWDE, i.e., chitinase) and siderophores, whereas *S. cavourensis* and *M. tulbaghiae* were associated with antibiotic and CWDE production, respectively. In greenhouse experiments the isolates showed high levels of disease protection in pre-inoculated mango seedlings, with a reduced number of defoliated leaves and lower counts of *L. theobromae* conidia (Kamil et al.).

Isolate S37 of *S. anulatus* from the rhizosphere of healthy *Vitis vinifera* promoted grapevine growth and induced resistance against *B. cinerea*, one of its most severe diseases. Interactions with S37, before and after the challenge with *B. cinerea*, induced local defense events including early responses such as oxidative burst, extracellular alkalinization, and activation of protein kinases, with expression of defense genes and phytoalexin accumulation. Moreover, Ca^2+^ also appeared to contribute upstream to the induced reactions. S37 primed grapevine cells for enhanced defense, with a decline in cell death rates. Grapevine cells also showed a distinct perception of beneficial and pathogenic microbes, as shown by desensitization assays based on alkalinization of extracellular pH. In fact, once the pH was increased by *S. anulatus* S37, the cells became refractory to further stimulation by *B. cinerea* (Vatsa-Portugal et al.).

## Bacterial Diseases

Takishita et al. tested a *Pseudomonas* sp. strain against *Clavibacter michiganensis* subsp. *michiganensis*, the causal agent of tomato bacterial canker. The strain activated a systemic resistance reaction in treated plants, as shown by marker genes such as PR1a and ACO. The observed reduction of *C. michiganensis* subsp. *michiganensis* cells was also linked to a better plant performance. The antagonist showed ability to solubilize inorganic phosphorus, and to produce siderophores, indole acetic acid, and hydrogen cyanide. These metabolic profiles, but hydrogen cyanide production, were also found in a strain of *Pseudomonas aeruginosa* active against *Xanthomonas oryzae* pv. *oryzae*, the causal agent of the bacterial leaf blight of Basmati rice, a severe disease in Pakistan. Yasmin et al. showed a significant pathogen suppression by the tested strain, likely mediated by secondary metabolites such as siderophores, rhamnolipids, and hydroxy-quinolines, underpinning an antibacterial activity. Moreover, the bacterium was capable of colonizing the rice rhizosphere, inducing the production of defense-related enzymes in treated plants, eventually increasing grain and straw yields by >50%.

## Management of Nematode and Insect Pests

Being one of the most severe crop limiting factors present in soil, the interaction of plant-parasitic nematodes with the rhizosphere microbiome has been the object of many studies. Several microorganisms both affect and are influenced by soil and rhizosphere food webs, of which nematodes represent a key component. Rhizosphere microorganisms may indeed counterbalance the reproduction of many nematodes, and in some cases they are determinant in soil suppressiveness (Westphal and Xing, 2011; Giné et al., 2016). However, cropping practices, and in particular monoculture, may dramatically shift this balance toward nematodes, due to their high reproductive rates in the presence of a suitable host. Elhady et al. investigated how the soil microbiome affects invasion and reproduction of plant-parasitic nematodes, studying the root knot nematode *Meloidogyne incognita* and the root lesion nematode *Pratylenchus penetrans*. They tested the effect of a transplanted rhizosphere microbiome from other crops (i.e., soybean, maize, or tomato), and the impact of a plant's own microbiome, in comparison to that from a fallow soil. The microbiome from maize or soybean significantly reduced root invasion by *P. penetrans*. Similarly, maize and tomato rhizosphere microbiomes affected the invasion of tomato roots by both *P. penetrans* and *M. incognita*, compared to soybean or bulk soil. Moreover, *M. incognita* was best suppressed on tomato by the own tomato rhizosphere microbiome. Data showed that the microbiome species composition, as selected by the previous cropping cycle, affect the subsequent development of a parasitic nematode population in soil.

In root-nematode interactions, the effect of soil bacteria may be deployed at multiple levels, depending on their metabolism and rhizosphere colonizing capability. Liu et al. studied the regulation of the soybean cyst nematode *Heterodera glycines* by an isolate of *Klebsiella pneumoniae*, a bacterium otherwise known for its clinical importance. One isolate, however, acts as plant growth promoter and biocontrol agent of sheath and seedling rice blights. The isolate tested showed an effect on nematode parasitism and density, when used as a seed coating or in field assays. Pot assays with split roots showed systemic resistance induced by *K. pneumoniae* in treated plants. Increased expression for some defense genes such as *PR1, PR2*, and *PR5* (pathogenesis-related genes), as well as *PDF1.2* (plant defensin), was observed. Moreover, the isolate improved the development of roots through the combined action of nitrogen fixation, phosphate solubilization, and siderophore production.

The links between plants and soil microorganism likely arise as the result of functional, coevolutive adaptations beneficial for the organisms involved. In this perspective it is useful to examine factors such as nutrients or substrates, present in the rhizosphere. Escudero et al. investigated the interaction of the hyphomycete *Pochonia chlamydosporia* with chitosan, a chitin-derived product. The fungus has multiple behaviors in soil, as a specialized parasite of nematode eggs, a saprotroph, or a root endophyte (Manzanilla-López et al., 2013). Chitosan enhances *P. chlamydosporia* sporulation, production of extracellular enzymes and egg parasitism (Escudero et al., 2016). At low concentrations (up to 0.1 mg ml^−1^) it improved the fungal growth and did not affect chlamydospores viability and germination. Treatments increased tomato root and plant development in presence of both *P. chlamydosporia* and *Meloidogyne javanica*, but did not affect eggs parasitism, unless a highly suppressive (>70% prevalence in eggs) sterilized soil was used.

As many antagonists occur in soil at the same time, it is worth checking whether their combined application may yield synergic effects. Experimental data indicated, however, that this situation is difficult to observe in natural conditions. Imperiali et al. studied field application of beneficial rhizosphere organisms, inoculated alone or in combination at wheat seeding. Tested antagonists included *Pseudomonas* spp. arbuscular mycorrhizal fungi (AMF), and entomopathogenic nematodes (EPN). The beneficial organisms persisted in soil after their introduction, as confirmed by molecular identification and increased plant health and productivity. However, no difference was observed between combined and single applications. Best wheat survival was reported on plants exposed to a biotic stress such as infestation by the frit fly, *Oscinella frit*, an important pest of cereals, which was reduced in treatments with *Pseudomonas* and AMF. Inoculations with EPN displaced some other native EPN, but only on the short-term.

## Suppressive Soils

Soils showing presence of a pathogen with, however, a low disease insurgence on a susceptible crop, are known as suppressive. This trait is likely due to the beneficial, indigenous microorganisms (Schlatter et al., 2017). Suppressive soils may be found in extensive cropping systems or in areas with long-term cultivation. Durán et al. screened soils for suppressiveness against “take-all” disease caused by the fungus *Gaeumannomyces graminis* var. *tritici*, sampling extensive wheat fields managed by indigenous Mapuche communities in Southern Chile. *In vitro* growth inhibition tests allowed identification of putative soils suppressive for take-all disease. Suppressiveness was closely associated to the soil microbiome, being lost upon soil sterilization and then recovered by addition of a natural soil inoculum.

A review on Verticillium wilts, vascular diseases caused on annual crops and woody perennials by several species of the soil-borne fungus *Verticillium*, showed the potential of bacteria belonging to *Bacillus* and *Pseudomonas* and of non-pathogenic xylem-colonizing *Verticillium* and *Fusarium* isolates as biocontrol tools. Soils and composts suppressive to Verticillium wilt allowed isolation of biocontrol agents characterized by useful traits such as inhibition of primary inoculum germination, plant growth promotion, competition, and induced resistance. However, *in vitro* antibiosis and mycoparasitism did not correlate with *in vivo* or *in planta* activities. Most useful traits included activity against the pathogen microsclerotia, a significant xylem and/or cortex colonization with nutrients and/or space competition, the induction of resistance reactions, and growth promotion in treated plants. All these effects should be screened under field conditions for selection of biocontrol agents suitable for large scale production, stable formulation, and application (Deketelaere et al.).

However, the impact on soil microbial communities of external factors such as the farmers' technical decisions (i.e., selecting crop species with/without rotations) should also be evaluated. A long-term monoculture increased the diversity of *F. oxysporum* and had a negative effect on antagonistic *Pseudomonas* spp. as shown by a 3 years long cropping of the medicinal plant *Radix pseudostellariae* (Chen et al.).

## Soil Environment

Several physical and climatic factors affect the microbiome diversity and the interactions of soil antagonists with plants, pests, and pathogens (Lareen et al., 2016). Mavrodi et al. characterized the establishment and activity of microbial communities in the rhizosphere of dryland wheat, and the microbiome response to irrigation. They also studied the population dynamics and activity of indigenous rhizobacteria producing antibiotics (phenazines), contributing to the natural suppression of wheat soilborne pathogens. Irrigation and soil moisture had a negative effect on plant colonization, density of bacteria and antibiotic levels. Although irrigation had a limited effect on the global microbiome diversity, differences were found for some groups such as the plant growth-promoting *Bacteroidetes* and *Proteobacteria*.

Atmospheric CO_2_ is a further physical factor related to climate change. Williams et al. studied its effect on strains of *Pseudomonas simiae* and *P. putida* in the rhizosphere of *Arabidopsis*. Differences in rhizosphere colonization were observed for the two bacteria, with an increase linked to CO_2_ levels (from 200 to 1,200 ppm), observed only for *P. simiae* in soil with relatively low C and N. Plant growth and systemic resistance to *P. simiae* varied in relation to the CO_2_ concentrations and soil type. Growth promotion and induced susceptibility were observed at sub-ambient CO_2_, whereas an opposite response was found at highest levels.

Stereoisomers of 2,3-butanediol, produced by some root-associated bacteria, were reported as triggering immunity of pepper plants against *Cucumber mosaic virus* and *Tobacco mosaic virus*. In a field trial, treatments with an enantiomer (2R,3R-butanediol) and a meso compound (2R,3S-butanediol) significantly reduced the prevalence of naturally occurring viruses, compared with a further enantiomer (2S,3S-butanediol) and control. Moreover, 2R,3R-butanediol induced the expression of plant defense genes in the ethylene/jasmonate and salicylic acid pathways at levels similar to those of benzothiadiazole-treated control (Kong et al.).

## Outlook and Future Challenges

Soil microbial communities offer an unlimited potential to develop locally customized and innovative approaches, facing major threats for global food security (Sergaki et al.). Challenges mainly concern plant protection and soil fertility. Many microorganisms that mineralize insoluble phosphate hold potential to improve growth and yield of many crops, in a sustainable way. This technology appears ready for commercial exploitation in various regions of the world, but more detailed information is still needed to be considered as an effective replacement of conventional fertilizers (Alori et al.). Studies on induced resistance and chemical communication of roots with soil microorganisms open unlimited perspectives for development of sustainable management tools, as shown in the reviews by Mhlongo et al. and Liu et al. Due to the many links among below- and above-ground physiological processes, chemical communication has been only partially deciphered, thanks to advanced techniques such as mass spectrometric-based metabolomics. The complexity and stability of microbial communities also depend on the capacity of roots to screen and select rhizosphere/rhizoplane taxa, mostly dominated by Proteobacteria with favorable traits such as motility, plant cell-wall degradation and ROS scavenging, nutrients procurement, nitrogen fixation, and defense priming.

## Author Contributions

AC wrote the manuscript. JM-B and CP revised the manuscript, helped structure and edit it, and approved its final version for publication.

### Conflict of Interest Statement

The authors declare that the research was conducted in the absence of any commercial or financial relationships that could be construed as a potential conflict of interest.
